# Aberrant regulation of FBW7 in cancer

**DOI:** 10.18632/oncotarget.1859

**Published:** 2014-03-25

**Authors:** Lixia Wang, Xiantao Ye, Yueyong Liu, Wenyi Wei, Zhiwei Wang

**Affiliations:** ^1^ The Cyrus Tang Hematology Center, Jiangsu Institute of Hematology, the First Affiliated Hospital, Soochow University, Suzhou, China; ^2^ Department of Pathology, Beth Israel Deaconess Medical Center, Harvard Medical School, MA, USA

**Keywords:** cancer, tumor suppressor, FBW7, SCF, ubiquitination, oncoprotein

## Abstract

FBW7 (F-box and WD repeat domain-containing 7) or Fbxw7 is a tumor suppressor, which promotes the ubiquitination and subsequent degradation of numerous oncoproteins including Mcl-1, Cyclin E, Notch, c- Jun, and c-Myc. In turn, FBW7 is regulated by multiple upstream factors including p53, C/EBP-δ, EBP2, Pin1, Hes-5 and Numb4 as well as by microRNAs such as miR-223, miR-27a, miR-25, and miR-129-5p. Given that the Fbw7 tumor suppressor is frequently inactivated or deleted in various human cancers, targeting FBW7 regulators is a promising anti-cancer therapeutic strategy.

## INTRODUCTION

FBW7 (F-box and WD repeat domain-containing 7), also known as Fbxw7, has been found to be involved in numerous cellular processes including cell proliferation, apoptosis, cell cycle and differentiation [1-3]. It has been well documented that human FBW7 encodes three transcripts: isoform α, β and γ, generated by alternative splicings. All three isoforms contain conserved interaction domains in the C-terminus and various isoform-specific domains in the N-terminal region [4]. Interestingly, these α, β and γ isoforms display distinct cellular localization patterns, in the nucleoplasm, cytoplasm and nucleolus, respectively [5]. Importantly, FBW7 is considered as a tumor suppressor protein in large due to the fact that FBW7 targets multiple well-known oncoproteins including Cyclin E [2, 6-9], c-Myc [10-13], c-Jun [14-16], Mcl-1 [17-19], and Notch-1[20, 21] for ubiquitination-mediated destruction. Consistent with the notion that FBW7 exerts its anti-tumor activity in various human malignancies, FBW7 mutation and/or deletion are frequently identified in a variety of human neoplasms [22]; for example, FBW7 mutation rate in T-cell acute lymphoblastic leukemia is approximately 30% [22].

Although recent studies have identified various downstream ubiquitin targets for FBW7, relatively little is known about the upstream signaling pathways that control FBW7 stability and cellular functions. To this end, there are some critical emerging evidence demonstrating that FBW7 tumor suppressor functions could be governed by multiple genes as well as upstream cellular signaling pathways. For example, it has been demonstrated that FBW7 is an unstable protein that undergoes self-ubiquitination [23]. Furthermore, studies have shown that the Pin1 oncoprotein directly interacts with FBW7 in a phosphorylation-dependent manner and promotes FBW7 self-ubiquitination and protein degradation [23]. Moreover, microRNAs (miRNAs) including miR-27, miR-25 and miR-223 have been reported to be involved in regulating the expression of FBW7 [24-27]. Therefore, in the following paragraphs, we will briefly summarize the newly identified substrates of FBW7 that have been reported in recent years that help further understanding the tumor suppressor role of FBW7. More importantly, we will mainly discuss how upstream genes and signaling pathways as well as miRNAs are involved in the regulation of the expression and stability of FBW7 to influence tumorigenesis.

### 2 The new downstream substrates of FBW7

Many studies from different groups have identified a growing list of specific substrates of FBW7 such as Aurora A [28], Cyclin E [8], c-Myc [29], c-Jun [14, 16], c-Myb [30-32], HIF-1α (Hypoxia inducible factor-1α) [33, 34], KLF5 (Kruppel-like factor 5) [35, 36], Mcl-1 (Myeloid cell leukemia-1) [18, 19], mTOR (mammalian target of rapamycin) [37, 38], NF1 (Neurofibromatosis type 1) [39], Notch [40, 41], NRF1 (Nuclear factor E2-related factor 1) [42], JUNB [43, 44], and SREBP (Sterol regulatory element-binding proteins) [45, 46]. Recently, multiple new targets of FBW7 including MED13 (Mediator 13), KLF2 (Krüppel-like factor 2), NF-κB2 [47, 48], and G-CSFR (Granulocyte colony stimulating factor receptor) [49] have been also discovered. Since several excellent review articles have already summarized the roles of FBW7 in human cancers [20, 22, 50, 51], we will briefly discuss these newly identified FBW7 substrates that would help us to further understand the critical role of FBW7 in tumorigenesis.

#### 2.1 Mediator 13 (MED13)

The Mediator complex is a multi-subunit complex that is required for active transcription by RNA polymerase II [52, 53]. It is known that the Mediator complex consists of 26 different subunits in yeasts. The Mediator complex has been identified in mammals as well. Moreover, a specific module, also known as the kinase module or the CDK8 module, has been found in some of the MED complexes [54]. Interestingly, the smaller core Mediator without the kinase module has been reported to have a stimulatory effect on transcription, while the larger form including both the core and the kinase module exerts repressive effect on a subset of genes, suggesting that the kinase module could be a key regulatory factor to govern the transcription activity [54]. For example, the CDK8 module reversibly associated with the Mediator core complex to control the Mediator-RNA Pol II interaction, leading to the regulation of transcription initiation and re-initiation. MED13 is the critical subunit for CDK8 sub-module-dependent repression. Recent studies have demonstrated that Mediators exert their functions largely through interacting with and coordinating the action of many transcriptional co-activators or co-repressors [52]. For example, MED15 has been shown to be targeted by the Smad2/4 transcriptional activators to control the TGF-β signaling [55] and participate in the SREBP signaling [56] as well. Similarly, MED14 is required for Mediator-dependent activation of genes regulated by the glucocorticoid receptor [57], HNF4 (Hepatocyte nuclear factor 4) [58], and PPARγ (Peroxisome proliferator-activated receptor gamma) [59]. However, ubiquitination of transcription factors has been implicated in either activating or terminating their activities [60, 61], but it is unclear whether the ubiquitination pathway controls the activity of Mediators.

To this end, a recent study has demonstrated that FBW7 could control the stability of MED13 [62]. Davis et al. revealed that FBW7 regulates CDK8 module-Mediator interactions and targets MED13 and MED13L for proteasomal degradation. Specifically, FBW7 interacts with the CDK8-Mediator complex [62]. Moreover, FBW7 was found to bind with several Mediator components including MED13. Notably, both MED13 and MED13L contain the optimal conserved phosphor-degron (CPD) consensus motif, through which FBW7 binds to its substrates [62]. To determine whether MED13/13L bound to FBW7, immunoprecipitation assays were conducted which showed that MED13/13L co-precipitated with FBW7 after proteasomal inhibition either by its inhibitor bortezomib or by using dominant-negative-Cullin1. Furthermore, the phospho-degron mutant form of Med13/13L (T326A mutation) failed to bind to FBW7 [62]. More importantly, MED13 and MED13L are phosphorylated at T326, which was shown to be required for FBW7-mediated degradation function [62]. Therefore, MED13 and MED13L are identified as *bona fide* FBW7 substrates. These important findings further suggested that FBW7 represents a novel mechanism for regulation of the Mediator activity to possibly influence the whole transcriptome [62]. However, additional in-depth investigation is required to elucidate whether under physiological or pathological conditions, the tumor suppressor role of FBW7 is exerted in part via regulating the Mediator complex.

#### 2.2 Kruppel-like factor 2(KLF2)

Krüppel-like factors (KLFs), members of the zinc finger family of transcription factors, have been found to be involved in the regulation of many biological processes, such as cell growth and differentiation [63, 64]. Accumulated evidence has suggested that KLFs play a critical role in tumorigenesis [65-67]. For example, KLF2 has been shown to exert cell growth-inhibitory, pro-apoptotic and anti-angiogenic functions. Consistent with this notion, KLF2 expression is diminished in a variety of human malignancies including prostate, breast, and ovarian cancers, suggesting that KLF2 may be a tumor suppressor gene [63]. Multiple studies have demonstrated that KLF2 could be regulated by upstream signaling pathways. For example, EZH2 (Enhancer of Zeste Homolog 2) was found to be one of the prominent genes to inhibit the expression of KLF2 [68]. Consistently, prostate or breast cancer patients with low expression of KLF2 and high levels of EZH2 have a shorter overall survival [68]. Additionally, Xie et al. reported that the Smurf1 (Smad ubiquitination regulatory factor 1) ubiquitin ligase targets KLF2 for ubiquitination and proteasomal degradation, leading to the regulation of its downstream genes such as CD62L and Wee1 in human cancer cells [69].

Recently, Wang et al. revealed that KLF2 is a substrate of FBW7, suggesting that FBW7 may regulate its biological functions through the destruction of KLF2 [70]. Specifically, two putative CPD sites on KLF2 were identified. In keeping with this finding, overexpression of FBW7 decreased KLF2 levels and shortened KLF2 protein half-life [70]. Furthermore, FBW7 interacted with KLF2 in the nucleus and promoted ubiquitination of KLF2. More importantly, GSK3β (glycogen synthase kinase 3 beta) was identified as a upstream kinase to specifically phosphorylate KLF2 on the T243 site within the identified phospho-degron [70]. Consistently, inhibition of GSK3β increased the basal levels of KLF2 and extended KLF2 protein half-life [70]. Taken together, KLF2 is a physiological substrate of FBW7, and further research in this area could provide insights into identification of agents that aim to suppress KLF2 or for targeted therapeutic treatments in FBW7-deficient cells.

**Figure 1:**
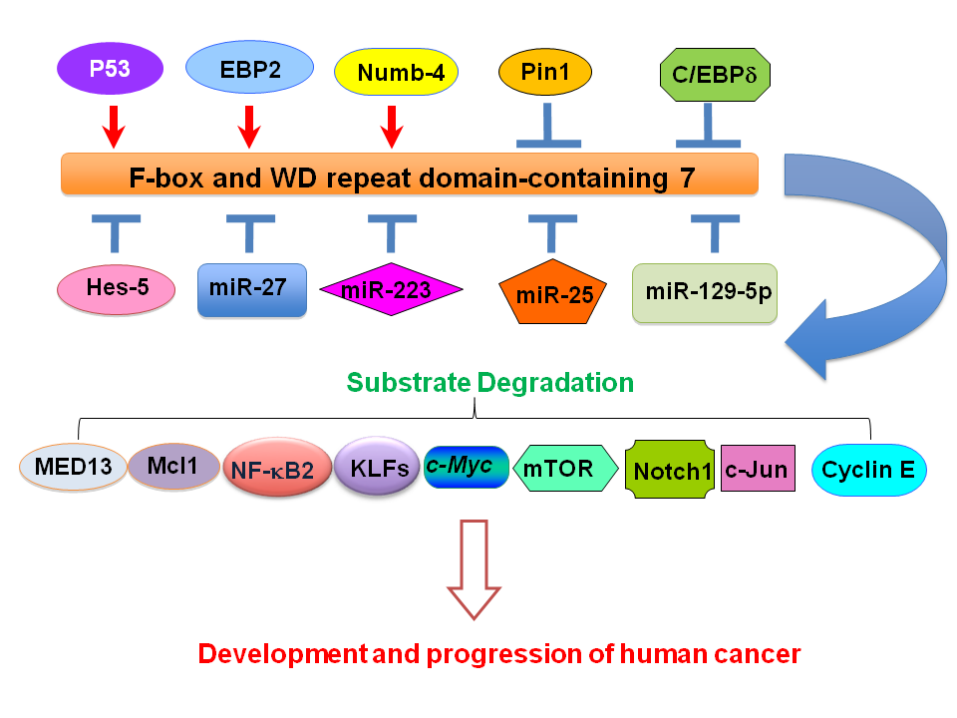
Illustration of upstream regulators that govern FBW7 expression in cancer Several upstream genes including p53, EBP2, Hes-5, Numb4, Pin1, and C/EBP-δ are reported to regulate FBW7 expression. In addition, multiple microRNAs (miRNAs) such as miR-27a, miR-25, miR-129-5p, and miR-223 have also been demonstrated to regulate the expression of FBW7. C/EBP-δ: CCAAT/enhancer-binding protein-δ; EBP2: Epstein-Barr nuclear antigen 1-binding protein 2; FBW7: F-box and WD repeat domain-containing 7; Hes-5: Hairy and Enhancer-of-split homologues 5; KLFs: Krüppel-like factors; Mcl-1: Myeloid cell leukemia-1; MED13: Mediator 13; mTOR: mammalian target of rapamycin; NF-kB2: Nuclear factor-κB2; Pin1: Peptidyl-prolyl cis-trans isomerase NIMA-interacting 1.

#### 2.3 Nuclear factor-κB2 (NF-κB2)

The NF-κB pathway has been well characterized to play important roles in the processes of development and progression of human cancers [71-75]. The NF-κB family is mainly composed of five proteins: RelA (p65), RelB, c-Rel, NF-κB1 (p50), and NF-κB2 (p52) [76]. NF-κB1 is sequestered in the cytoplasm through association with its inhibitors: IκBα and p100 proteins. It is known that p100 also serves as precursor of NF-κB2 DNA-binding subunits [71]. The NF-κB is activated after it is dissociated from its inhibitors, and subsequently translocates to the nucleus for activation of NF-κB target genes [76, 77]. It is clear that IκBα and p100 could be phosphorylated by IKKβ and IKKα, respectively, resulting in the degradation of IκBα by the 26S proteasome and the processing of p100 into smaller forms (p52) [73]. NF-κB has been found to be critically involved in tumorigenesis, exerting its oncogenic roles through the regulation of cell proliferation, differentiation, apoptosis, migration, invasion and angiogenesis [76, 78].

Three independent groups recently identified that NF-κB2/p100 is a substrate of FBW7 [47, 48, 79]. Notably, FBW7 interacts with p100 via a conserved phospho-degron. Moreover, FBW7 promoted degradation of p100, which was largely in a GSK3 phosphorylation-dependent manner [79]. Overexpression of FBW7 caused enhanced NF-κB activity in FBW7 null cells [47]. Consistently, FBW7 inactivation up-regulated p100 levels, which subsequently suppressed the canonical NF-κB1 signaling as p100 precursor could suppress NF-κB1 transcriptional activities in an IκB−like manner. Busino et al. further identified that the FBW7α isoform controls p100 degradation [48]. More importantly, this group found that FBW7α silencing led to the induction of cellular apoptosis in multiple myeloma cells and xenotransplant models in part through regulating p100 degradation [48]. These findings provide mechanistic insights for the regulation of NF-κB pathway by FBW7 through the destruction of NF-κB2/p100.

#### 2.4 Granulocyte colony stimulating factor receptor (G-CSFR)

G-CSFR, a critical regulator of granulopoiesis, has been reported to play a pivotal role in tumorigenesis [80-82]. To this end, it has been shown that G-CSF/G-CSFR, though both autocrine and paracrine mechanism, enhanced cell survival and promoted cell growth in bladder cancer cells [83]. Yang et al. revealed that G-CSFR was critically involved in the development and progression of human colorectal cancers [84]. This group observed that G-CSFR was up-regulated in the human colorectal cancer setting. Notably, G-CSFR expression was correlated with tumor stage and tumor differentiation [84]. In support of their finding, Kunter et al. observed that truncated G-CSFR cooperated with the PML-RARα (promyelocytic leukemia-retinoic acid receptor alpha) oncogene to induce acute myeloid leukemia (AML) in mice [85].

Interestingly, there is emerging evidence demonstrating the possible role of the ubiquitin/protease system in regulating G-CSFR expression [86]. Moreover, studies have demonstrated that ubiquitination is required for regulation of G-CSFR-mediated cell proliferation. G-CSFR mutations could disrupt its ubiquitination and subsequently cause aberrant receptor signaling, leading to leukemic transformation [87]. Recently, Lochab et al. found that FBW7 negatively controlled the granulocytic differentiation in part by targeting G-CSFR for degradation [49]. Furthermore, it has been shown that both FBW7 and GSK3β are required for G-CSFR degradation. More importantly, FBW7-mediated destruction of G-CSFR suppressed STAT3 (signal transducer and activator of transcription 3) phosphorylation and activation [49]. In line with this finding, inhibition of FBW7 restored G-CSFR signaling and subsequently increased STAT3 transcriptional activity. Interestingly, FBW7 also interacts with and degrades a truncated mutant of G-CSFR, G-CSFR-T718, which was frequently found in AML [49]. Overall, these reports suggest that G-CSFR could be a substrate of FBW7 and aberrant upregulation of G-CSFR due to impairments in FBW7-mediated destruction could contribute to the development of AML.

### 3 Regulation of FBW7

It is worth mentioning that most studies focus on discovering the ubiquitin targets of FBW7 ubiquitin ligase pathway. However, how FBW7 itself is regulated is largely unclear in human cancers. To this end, emerging evidence has demonstrated that several molecules such as p53, Pin1 (Peptidyl-prolyl cis-trans isomerase NIMA-interacting 1), C/EBP-δ (CCAAT/enhancer-binding protein-δ), Hes-5 (Hairy and Enhancer-of-split homologues 5), Numb, as well as microRNAs (miRNAs) including miR-27a and miR-223 have been found to regulate the expression of FBW7 [88-91]. In the following section, we will discuss the mechanisms how FBW7 is controlled by its upstream regulators.

#### 3.1 Regulation of FBW7 by p53

It has been well documented that p53 functions as a major tumor suppressor protein in the majority of human cancers [92-95]. The p53 tumor suppressor is found to be involved in cell growth, DNA synthesis and repair, differentiation, apoptosis, and cellular responses to a wide range of cellular stresses including heat shock, hypoxia, osmotic shock and DNA damage [96, 97]. Since p53 mutation/deletion has been identified in, at least, 50% of all human cancers, cancers with a p53 mutation/deletion generally have bad prognosis due to poor response to therapeutics [98]. Previous studies have shown that p53 is negatively regulated by Mdm2, which is an E3 ubiquitin ligase that promotes the ubiquitination and destruction of p53 [99]. In support of this notion, Nutlin-3, a well-characterized Mdm2 antagonist, can bind to Mdm2 in the p53-binding pocket and interfere with Mdm2-mediated p53 degradation, leading to the accumulation of p53 [100]. Further research has shown the effects of novel Mdm2 inhibitors in maintaining p53 function [101, 102].

Recently, FBW7 has been identified as a direct *bona fide* transcriptional target of p53 [88, 103]. Kimura et al. initially found that the expression of FBW7 was dramatically up-regulated by infection with adenovirus-mediated transfer of wild-type p53 into the p53-deficient cells [88]. Moreover, they further demonstrated that the first exon of FBW7 contains a p53-binding site that has p53-dependent transcriptional activity. Furthermore, expression of FBW7β was induced in a p53-dependent manner after genotoxic stress such as UV irradiation, suggesting that FBW7 is a direct target of p53 [88]. Keeping abreast with this finding, Mao et al. subsequently reported that FBW7 mediates the critical role of p53 in response to DNA damage, indicating that FBW7 gene is a p53-dependent tumor suppressor gene involved in tumorigeneis [89]. Furthermore, this group found that inhibition of mTOR signaling pathway by rapamycin after exposure to radiation retarded tumor development in FBW7/p53 double heterozygous mice [104]. Taken together, these studies showed that targeting the p53 signaling pathway could potentially influence FBW7 expression, which might provide a feasible approach to restore FBW7 expression for anti-cancer therapies.

#### 3.2 Regulation of FBW7 by C/EBP-*δ*

C/EBP-δ is one of six isoforms of the C/EBP family that is a highly conserved family of leucine zipper type DNA-binding proteins [105, 106]. To exert its biological function, C/EBPs need to form homodimers or heterodimers with other C/EBP family members as well as other transcriptional factors including c-Fos and NF-κB (nuclear factor-κB) [105]. C/EBP-δ has been found to be involved in the regulation of growth and differentiation of a variety of cell types [107]. For example, forced expression of C/EBP-δ inhibited prostate cancer cell growth [108], suggesting a tumor suppressor function for C/EBP-δ. Moreover, it has been reported that C/EBP-δ initiates and maintains mammary epithelial cells at the G0 growth arrest. Furthermore, reduced C/EBP-δ gene expression due to promoter methylation has been found in breast cancer cell lines and primary breast tumors [109].

Recently, Pawar et al. reported that C/EBP-δ induced expression of the Cdc27 subunit of the APC/C (anaphase promoting complex/cyclosome), leading to the degradation of the Cyclin D1 and other APC substrates Cyclin B1, Skp2 and Plk-1 [110]. More recently, Roysarkar et al. identified a novel pathway by which Src suppressed C/EBP-δ in part through the SIAH2 (Seven in absentia homolog 2) E3 ubiquitin ligase [111]. Furthermore, it was recently reported that C/EBPδ can directly inhibit the expression of FBW7 [90]. This study demonstrated that C/EBPδ enhanced the mTOR/Akt/S6K1 signaling and augmented the translation and activity of HIF-1α through inhibiting FBW7 as both mTOR and HIF-1α are reported substrates of FBW7 [90]. Surprisingly, this study also revealed that C/EBPδ promoted breast tumor metastasis [90], indicating that further investigation is required to determine the molecular mechanisms, especially the contribution of FBW7 in mediating the cellular function of C/EBPδ in promoting tumor metastasis.

#### 3.3 Regulation of FBW7 by EBP2

Recently, Welcker et al. have identified that the EBNA1 (Epstein-Barr nuclear antigen 1)-binding protein 2 (EBP2) directly binds to FBW7 and regulates FBW7's nucleolar localization [112]. It is known that EBP2 is a small nucleolar protein that is essential for mammalian cell proliferation [113]. However, the exact role of EBP2 in tumorigenesis is largely unknown. The study led by Dr. Clurman demonstrated that EBP2 serves as a pseudo-substrate to target FBW7 to the nucleoli compartment [112]. Hence, it is important to further discuss the special role of EBP2 as a pseudo-substrate for FBW7. First, EBP2 contains a canonical CPD motif at its extreme N terminus [112]. Second, EBP2 binding to FBW7 is regulated by phosphorylation of EBP2 by GSK3 [112]. Third, FBW7 binding to EBP2 is uncoupled from the regulated turnover of endogenous EBP2, arguing that FBW7 binding to endogenous EBP2 appears insufficient for the degradation event of EBP2 *in vivo* [112]. Fourth, inactivation of FBW7 did not cause any noticeable changes to neither endogenous EBP2 abundance nor EBP2 stability [112]. Taken together, similar to SV40 T antigen [114], EBP2 behaves mostly like the FBW7 pseudo-substrate that mainly serves to mediate its nucleolar localization.

#### 3.4 Regulation of FBW7 by Pin1

It has been well documented that Pin1 is the only enzyme known that can isomerize specific Ser/Thr-Pro peptide bonds after phosphorylation to regulate their conformational changes with high efficiency [115-117]. These Pin1-induced conformational changes could regulate protein stability, catalytic activity, phosphorylation status, protein-protein interactions, and subcellular localization to further impact a wide range of cellular processes [118, 119]. Because regulating these protein functions by Pin1 is involved in diverse physiological and pathological processes, Pin1 deregulation is implicated in a number of diseases, including aging and age-related diseases, such as Alzheimer disease and cancer [120]. For example, Pin1 is overexpressed in most human cancers. Notably, Pin1 overexpression is associated with poor clinical outcomes in human cancer patients. Accumulated evidence has demonstrated that Pin1 exerts its oncogenic functions in large through activation of numerous oncogenes including Neu, Ras, c-Jun, Mcl-1, Notch-1, c-Myb, and inactivation of a large number of tumor suppressors such as p53, PML, and Foxos [121-123].

Recent studies have shown that Pin1 regulates the stability of several FBW7 substrates such as Mcl-1 and c-Jun [124], indicating that Pin1 may be an upstream regulator of FBW7. Indeed, Min et al. found that Pin1 directly binds to FBW7 and disrupts FBW7 dimerization [23]. Specifically, Pin1 interacts with FBW7 in a phosphorylation-dependent manner [23]. Moreover, Pin1 negatively regulated the stability of FBW7 through promoting FBW7 self-ubiquitination and degradation [23]. Mechanistically, Pin1 inhibited FBW7 dimerization, which is one of the key regulatory mechanisms for the regulation of FBW7. Furthermore, over-expression of Pin1 showed reduced FBW7 protein abundance, leading to the inhibition of tumor cell proliferation and transformation [23]. Consistently, depletion of Pin1 caused higher expression of FBW7, subsequently decreased Mcl-1 abundance, leading to enhanced Taxol sensitivity in cancer cells [23]. These results suggest that Pin1 reduces FBW7 expression, and thereby facilitating tumorigenesis. Taken together, this study provides the rationale for the development of specific Pin1 inhibitors as potential anti-cancer agents.

#### 3.5 Regulation of FBW7 by Hes-5

Notch signaling pathway has been known to regulate various cellular processes including cell proliferation, apoptosis, migration, invasion, and angiogenesis in human malignancies [125-127]. Notch pathway is a ligand-receptor pathway with four characterized Notch receptors (Notch-1, 2, 3, 4) and five reported ligands (Dll-1, 3, 4 and Jagged-1, 2) [128, 129]. The Notch pathway is activated when Notch ligand binds to its receptor. Notch intracellular domain (NICD) is produced by the cleavage of membrane-bound Notch by multiple enzymes and released into the cytoplasm, subsequently translocates to the nucleus and activates its target genes including Hes-1, Hes-5, Hey-1, etc [128]. It has been well documented that Notch signaling pathway contributes to tumor development and progression [130-132]. Over-expression of Notch receptors and ligands, and their target genes has been found in a variety of human cancers [133-136]. For example, the expression of Hes-1 and Hes-5 are up-regulated in advanced ovarian serous adenocarcinomas [137]. Notably, Hes-1 high expression could be a potential poor prognostic factor for ovarian cancer patients [137]. In line with this notion, Hes-1 and Hes-5 expressions were significantly higher in squamous cervical carcinomas [138]. Moreover, Hes-1 and Hes-5 are positively associated with various prognostic factors in early-stage cervical carcinoma, suggesting that both Hes-1 and Hes-5 could be useful biomarkers to predict poor prognosis in patients with cervical carcinoma [138]. Study from Sancho and coworkers has demonstrated that Hes-5 directly represses transcription of FBW7β [139]. Furthermore, they revealed that the NICD/Hes-5/ FBW7β positive feedback loop underlies FBW7 haploinsufficiency [139]. However, further in-depth investigation is required to explore mechanistically how Hes-5 inhibits FBW7 expression.

#### 3.6 Regulation of FBW7 by Numb4

Numb is originally found to be required for cell fate determination during the neuroblast division [140, 141]. Recently, Numb has been identified as a *bona fide* tumor suppressor gene in human cancers [142]. In breast tumors, frequent loss of Numb expression was observed and it was correlated with poor prognosis [143]. Similarly, the expression of Numb is frequently lost in NSCLC (non-small cell lung carcinomas) [144]. Consistently, Numb deletions and low Numb expression have also been observed in pro-neural glioblastomas [145]. Numb has been reported to regulate multiple signaling pathways such as p53, Notch, and Hedgehog [142]. Study has suggested that Numb binds to and inhibits the E3 ubiquitin ligase Mdm2, which is responsible for p53 degradation, subsequently leading to up-regulation of p53 [146, 147]. Numb is able to bind the Itch, an E3 ubiquitin ligase, leading to the ubiquitination of Notch, suggesting that Numb could act as an adapter between Notch and Itch [148, 149]. It should be noted that Numb itself could be regulated by the E3 ligases including Siah-1 and LNX, resulting in its ubiquitin-dependent degradation [150, 151]. Recently, it has been shown that one of the predominant Numb isoform, Numb4, promoted FBW7 ubiquitin ligase assembly and activation, leading to enhanced Notch degradation [145]. However, further in-depth investigation is warranted to understand the physiological contribution of Numb4-mediated regulation of FBW7 expression in tumorigenesis *in vivo*.

#### 3.7 Regulation of FBW7 by the microRNAs (miRNAs)

In recent years, miRNAs have been demonstrated to regulate gene expression through binding to the 3'UTR (3' untranslated region) of target mRNAs, leading to either inhibition of translation of the encoded proteins or destabilization of the target mRNAs [152, 153]. It is well characterized that miRNAs could exert their oncogenic or tumor suppressor functions depending on the various target genes they control [154-156]. Accumulated evidence has also shown that multiple miRNAs including miR-27 and miR-223 could regulate FBW7 expression [24, 91]. In the following paragraphs, we will discuss the potential function of miRNAs that are involved in the regulation of FBW7 to influence it anti-tumor roles.

##### 3.7.1 Regulation of FBW7 by miRNA-27

It has been documented that miR-27a plays an oncogenic role in human cancers [157, 158]. For example, the oncogenic activity of miR-27a was observed in breast cancer cells due to the suppression of zinc finger ZBTB10, leading to increased expression of specificity proteins including Sp1, Sp3, and Sp4, and subsequently causing up-regulation and activation of Sp-dependent survival and angiogenic genes, such as Survivin, VEGF and VEGFR1 [159]. Consistently, another study demonstrated that miR-27a was highly expressed in breast cancer cells and inhibited the expression of tumor suppressor FOXO1 [160]. Zhu et al. found that miR-27a was critically involved in drug resistance through regulating P-glycoprotein and MDR1 (multidrug resistant) expression in human cancer cells [161]. Similarly, down-regulation of miR-27a reversed multidrug resistance of esophageal squamous cell carcinoma in part via regulation of P-glycoprotein, Bcl-2 and MDR1 [162]. In line with the oncogenic roles of miR-27a, Liu et al. reported that miR-27a is overexpressed in human gastric adenocarcinoma and promotes gastric cancer cell growth by inhibition of Prohibitin [163]. Furthermore, miR-27a exerts its oncogenic function via regulation of MET, EGFR, and Sprouty2 in lung cancer [164]. Ma et al. have further revealed that miR-27a enhanced cell growth, colony formation and migration by targeting Sprouty2 in pancreatic cancer cells [165]. Notably, miR-27a was found to regulate endothelial differentiation of breast cancer stem like cells [166]. Taken together, miR-27a could be possibly used as a target in the diagnosis and treatment of human cancers.

Recently, miR-27a was identified to inhibit the expression of FBW7. Wang et al. reported that FBW7 is a potential miR-27a target. Consistently, there is an inverse correlation between miR-27a expression and FBW7 levels in human tumor samples [167]. Lerner et al. further discovered that miR-27a suppresses FBW7 during specific cell cycle phases [26]. Specifically, miR-27a suppresses FBW7 expression, leading to a reduction in ubiquitin-mediated degradation and turnover of FBW7 substrate, cyclin E. Overexpression of FBW7 caused dysregulation of cyclin E, resulting in altered cell cycle progression [26]. Moreover, miR-27a was found to be overexpressed and inversely associated with FBW7 expression in leukemia [26]. In further support of this concept, overexpression of miR-27a has been observed in colon cancer cell lines and colon cancer stem cells [168]. Notably, miR-27a knockdown increased FBW7 levels and subsequently decreased the expression of FBW7 substrates such as c-Myc, c-Jun and Notch-1 in colon cancer [26]. Furthermore, miR-27a overexpression promotes cell growth, whereas miR-27a knockdown inhibits cell proliferation in vitro and tumor formation *in vivo* possibly through regulating FBW7 [26]. Collectively, these findings suggest that miR-27a exerts its oncogenic functions in part through negative regulation of the FBW7 tumor suppressor.

##### 3.7.2 Regulation of FBW7 by miR-223

Recent evidence has suggested that miR-223 may play a key role in human cancers [169-171]. Numerous studies have found overexpression of miR-223 in various types of human cancers including hepatocellular carcinoma [172], T-ALL [173], ovarian cancer [174], gastric cancer [25], esophageal squamous cell carcinoma [175], and bladder cancer [176]. Moreover, overexpression of miR-223 has been found in patients with lymph node metastasis and metastatic disease at an advanced pathological stage in gastric carcinoma [25]. High expression of miR-223 was also found to be associated with poor survival in gastric carcinomas [177], ovarian cancer [174], and esophageal squamous cell carcinoma [175]. Furthermore, miR-223 promotes gastric cancer invasion and metastasis by targeting tumor suppressor EPB41L3 [177]. Interestingly, miR-223 was found to have a tumor suppressor function by inhibiting migration and invasion through targeting Artemin, a tumor metastasis-related gene, in human esophageal carcinoma [178]. In line with this notion, it has been shown that miR-223 is overexpressed in the multiple step progression of Barrett's esophagus and modulates drug resistance via targeting PARR1 [179]. Interestingly, in a separate report, down-regulation of miR-223 was identified in hepatocellular carcinoma, while re-expression of miR-223 caused an inhibitory effect on cell viability in hepatocellular carcinoma cell lines [180]. Therefore, further study is warranted to determine the physiological function of miR-223a in various human tumor settings.

A growing body of evidence has recently demonstrated that miR-223 regulates FBW7 expression. Xu et al. found that the over-expression of miR-223 suppressed FBW7 expression, resulting in increased Cyclin E protein levels and activities, and subsequently causing genomic instability [24]. Conversely, reduced miR-223 expression resulted in increased FBW7 expression and decreased Cyclin E activity, indicating that FBW7 can be modulated directly by miR-223 [24]. Li et al. also reported that FBW7 protein levels were inversely correlated with miR-223 expression in gastric tumor tissues [25]. Re-introduction of miR-223 suppressed FBW7 expression at the post-transcriptional level in gastric cancer cell lines [25]. Consistent with this finding, Kurashige and colleague reported similar inverse relationship between miR-223 and FBW7 in esophageal squamous cell carcinoma [175]. This group also identified FBW7 as a functional downstream target of miR-223 in esophageal cancers [175]. Recently, Mavrakis et al. found that Mcl-1 levels are increased in mouse leukemias expressing miR-223 through down-regulation of FBW7 [181]. However, further in-depth research is needed in order to fully understand how miRNA-223 regulates FBW7 in human carcinogenesis.

##### 3.7.3 Regulation of FBW7 by miR-25

Several lines of evidence has defined that miR-25 is dysregulated in human cancers [182-184]. For example, miR-25 was observed to be down-regulated in human colon cancer tissue. Moreover, studies have shown that miR-25 could inhibit cell growth and migration through repression of Smad7 in colon cancer cells [185]. Another study showed that miR-25 could inhibit cell proliferation and colony formation through targeting oncogene EZH2 (enhancer of zeste 2) in thyroid carcinoma [186]. In contrast, miR-25 has been found to be up-regulated in esophageal squamous cell carcinoma (ESCC) tissues and associated with lymph node metastasis and TNM (Tumor, Node and Metastasis) stage. This study also revealed that miR-25 promoted ESCC migration and invasion via inhibiting E-cadherin expression [187]. Consistently, miR-25 promotes cell aggressiveness through down-regulation of desmpcollin-2 and subsequently redistributing adheren junctions and activating β-Catenin signaling in ESCC [188]. In addition, miR-25 has also been found to be highly expressed in ovarian cancer and regulates apoptosis through targeting pro-apoptotic Bim [189]. Additionally, miR-25 promotes apoptosis resistance through targeting TNF-related apoptosis inducing ligand (TRAIL) death receptor-4 in cholangiocarcinoma [190]. Recently, miR-25 has been shown to inhibit FBW7 expression and caused up-regulation of c-myc and KLF5 to promote reprogramming of mouse fibroblast cells to iPSCs (induced pluripotent stem cells) [191]. However, further studies are warranted to explore whether miR-25 regulates FBW7 in cancer stem cells in the future.

##### 3.7.4 Regulation of FBW7 by miR-129-5p

Mounting evidence suggested that miR-129-5p may also be involved in tumor development and progression [192, 193]. For example, it has been demonstrated that miR-129-5p is required for histone deacetylase inhibitor-induced cell death in thyroid cancer cells [194]. Liu et al. found that miR-129-5p suppressed tumor growth and reduced cell migration through inhibition of VCP (Valosin containing protein) in hepatocellular carcinoma [195]. Zhi et al. reported that miR-129-5p was up-regulated in serum in AML patients, indicating that miR-129-5p could be a potential biomarker for detecting AML [196]. Recently, miR-129-5p was identified to regulate FBW7 expression. Over-expression of miR-129-5p up-regulated FBW7 expression, however; the underlying mechanism is unclear [197]. Accordingly, further study is necessary to define how miR-129-5p controls FBW7 expression and whether this process is involved in tumorigenesis.

#### 3.8 Regulation of FBW7 by other factors

Emerging evidence has suggested that FBW7 could be regulated by several other factors such as NF-κB1 [198], FAM83D (family with sequence similarity 83, member D) [199]. For example, Huang et al. found that NF-κB1 inhibited FBW7 expression and subsequently suppressed its target c-Myc protein degradation [198]. Wang et al. reported that FAM83D promoted cell proliferation and migration as well as invasion through downregulation of FBW7 and upregulation of FBW7 target mTOR [199]. Another study showed that SREBP2 regulated miR-182 targeting FBW7, leading to a feedback pathway to regulate SREBP transcriptional activity [200, 201]. Moreover, various stress stimuli have been found to induce FBW7 expression [202]. Interestingly, various stress stimuli regulated different FBW7 isoforms. Specifically, FBW7β is induced by all the stress stimuli such as aphidicolin, fetal calf serum, vinblastine mostly in a p53-dependent manner, whereas expression of FBW7α and γ was responded to limited stress stimuli [202].

## CONCLUSIONS

In conclusion, FBW7 functions as a tumor suppressor, which is frequently mutated or depleted in various types of human cancers. FBW7 exerts its anti-tumor activity by targeting a ever-growing list of ubiquitin substrates including MED13, KLF2, and Mcl-1. More importantly, the E3 ligase activity of FBW7 is regulated by many upstream genes such as p53, C/EBP-δ, EBP2, Pin1, Hes-5 and Numb4. Moreover, miRNAs including miR-223, miR-27a, miR-25, and miR-129-59p can also regulate the expression of FBW7. Since FBW7 is governed by upstream factors, restoring FBW7 tumor suppressor function through regulation of these factors could be useful to design novel therapeutic means to treat cancer patients. Indeed, it has been demonstrated that a natural dietary agent genistein inhibited miR-223 expression and subsequently up-regulated FBW7, leading to cell growth inhibition and apoptosis in pancreatic cancer cells [203]. Therefore, targeting FBW7 upstream genes and its regulatory miRNAs could open a novel therapeutic window for developing more potent treatments of human cancers.
